# ContigExtender: a new approach to improving de novo sequence assembly for viral metagenomics data

**DOI:** 10.1186/s12859-021-04038-2

**Published:** 2021-03-12

**Authors:** Zachary Deng, Eric Delwart

**Affiliations:** 1 Vitalant Research Institute, San Francisco, CA 94118 USA; 2 Department of Laboratory Medicine, University of California at San Francisco, San Francisco, CA 94107 USA

**Keywords:** Metagenomics, De novo assembly, Next-Gen Sequencing, Viral discovery, Pathogen detection

## Abstract

**Background:**

Metagenomics is the study of microbial genomes for pathogen detection and discovery in human clinical, animal, and environmental samples via Next-Generation Sequencing (NGS). Metagenome de novo sequence assembly is a crucial analytical step in which longer contigs, ideally whole chromosomes/genomes, are formed from shorter NGS reads. However, the contigs generated from the de novo assembly are often very fragmented and rarely longer than a few kilo base pairs (kb). Therefore, a time-consuming extension process is routinely performed on the de novo assembled contigs.

**Results:**

To facilitate this process, we propose a new tool for metagenome contig extension after de novo assembly. ContigExtender employs a novel recursive extending strategy that explores multiple extending paths to achieve highly accurate longer contigs. We demonstrate that ContigExtender outperforms existing tools in synthetic, animal, and human metagenomics datasets.

**Conclusions:**

A novel software tool ContigExtender has been developed to assist and enhance the performance of metagenome de novo assembly. ContigExtender effectively extends contigs from a variety of sources and can be incorporated in most viral metagenomics analysis pipelines for a wide variety of applications, including pathogen detection and viral discovery.

**Supplementary Information:**

The online version contains supplementary material available at 10.1186/s12859-021-04038-2.

## Background

Metagenomic next-generation sequencing (mNGS) has emerged as an unbiased, high throughput tool for clinical infectious agent detection and novel pathogen discovery [1–6]. Analytical metagenome pipelines are currently undergoing active development [7–11]. To identify microbial sequences, millions of NGS reads are compared to publicly available databases of reference sequences. In this analysis, the creation of longer contigs from short overlapping reads is accomplished through de novo metagenome assembly. The longer contigs generated by high-quality sequence assembly have two major advantages over short reads: improved detection sensitivity of novel pathogens without strong sequence homology to known pathogens; and a reduced need of manual genome extension through polymerase chain reaction [12].

De novo assembly has been an essential tool in recent studies in metagenomics viral discovery [13–22]. Dedicated metagenome assemblers have emerged recently, as a result of very active development in this field. Several metagenome assemblers are summarized in a recent review by Ayling et al. [23] and tested in our previous study [12]. Recently, the popular assembler SPAdes and its variant, metaSPAdes, specially designed for metagenomic applications, have emerged to be the tools of choice for metagenome assembly [24, 25]. The metaSPAdes algorithm is based on De Bruijn graphs (DBG) and has addressed many challenges in metagenome assembly, such as uneven coverage and the existence of mixtures of multiple strains. Since metaSPAdes has been adopted by many labs, we will use it as our benchmark tool to generate the initial seed contigs to be extended. Other DBG-based metagenome assemblers include IDBA-UD [26], MetaVelvet [27], MetaVelvet-SL [28], MEGAHIT [29], MegaGTA [30], Ray Meta [31], PRICE [32], and Xander [33]. Another type of assembler employs a strategy called Overlap-Layout-Consensus (OLC), which is based on joining overlaps to form new contigs. This group includes SAVAGE [34], Snowball [35], Genovo[36], BBAP [37], IVA [38], and VICUNA [39].

According to previous data [8, 12], de novo assemblers, when applied to real metagenomic samples, are unlikely to produce contigs longer than several kb. Uneven coverage, sequencing errors, library construction, and amplification artifacts are among the factors causing premature assembly termination. Another challenge is the presence of multiple viral, bacterial, and animal/host DNA fragments within metagenomic samples. However, in practice, it is often possible to find reads that overlap with the edges of the de novo assembled contigs, so iterative extension may significantly increase contig length. We are motivated by this observation and propose a novel algorithm and a software tool, ContigExtender, to automate the contig extension step following de novo assembly.

The basic ContigExtender algorithm is a greedy algorithm based on overlap search, with the following steps: (1) find overlapping reads with respect to both ends of the input contig; (2) calculate candidate extension paths based on these overlapping reads; (3) extend the existing contig; and (4) repeat the process until it can no longer be extended. To ensure it works properly under challenging situations, ContigExtender has several important unique features. First, the extension path is allowed to branch when multiple extension paths representing multiple strains are present. This is implemented using depth-first search (DFS) to explore multiple possible extending paths recursively. The benefit of this feature is to avoid the extension becoming trapped in local optima, which may cause pre-mature termination. Second, the algorithm focuses on overlapping quality rather than depth. A hard depth cutoff is avoided to allow contig extension in ultra-low coverage (1×) but otherwise high-quality overlapping areas. Last, instead of using a consensus sequence derived from all covering reads at the overlap, we separate the region into genotypes, since the overlapping reads may contain multiple strains. Overlapping reads are categorized according to their genotypes and the genotypes are ranked based on read concentration and quality. The main benefit of using genotypes instead of a single consensus is that it allows for branching for each strain. In addition, untrimmed adapters and sequencing errors are not likely to contribute to significant genotypes since these contaminations appear at random positions.

De novo assembler generated contigs are *seed contigs* that are input into ContigExtender. The outputs of ContigExtender are *final contigs*. The final contigs were rigorously evaluated based on *gained length* and *contig accuracy*, which is measured by similarity to NCBI viral reference nucleotide sequences using nucleotide BLAST [40]. Our results show that in comparison to other approaches to contig extension, ContigExtender is effective at extending seed contigs while maintaining high levels of contig accuracy.

## Implementation

### Datasets

Four groups of datasets, named “in silico synthetic”, “NIBSC virus standard”, “Animal”, and “Human”, were used to evaluate the ContigExtender algorithm. To evaluate performance (contig size and accuracy), we compared the output to the reference genome of the target pathogen.

A proof of concept “in silico synthetic” dataset was developed to test the efficacy of ContigExtender at varying read lengths (100 bp, 250 bp), error rates (1% and 5%), depth (10x, 20x, and 50x), and unevenness of coverage. Three target viral genomes include the Bas-Congo virus (BASV), a novel rhabdovirus associated with hemorrhagic fever cases in central Africa [41]; BK virus (BKV), a human polyomavirus; and human immunodeficiency virus type 1 (HIV-1). BKV is an unenveloped double-stranded DNA virus with circular genomes of around 5kbp. BASV genome is a negative-sense single-stranded RNA virus and HIV-1 is a positive-sense single-stranded RNA virus.

To emulate the unevenness of coverage, peaks of 50× coverage spanning 250 bp were spiked-in every 1 kb. Each genomic position has equal probability to be covered, emulating the ideal shot-gun sequencing process. However, the stochastic nature of this process will not result in perfect even coverage across the genome, but rather a binomial coverage distribution, especially in a low coverage situation. For example, we have observed that some positions can have as low as 1× coverage while the average coverage is 10x.

In addition, we simulated realistic 100 bp paired-end Illumina reads using pIRS version111 [42] for the above three reference viruses. This software does not allow longer reads, but it considers GC content, derived from real Illumina base calling profiles.

The NIBSC, Animal and Human samples are summarized in Table 1. The NIBSC dataset [43] (NIBSC sample 26) is assembled from both clinical specimens and cultured viruses. The target viral genomes range in length from ~ 6 to ~ 234 kb. Illumina MiSeq sequencing created a dataset of approximately 9 million paired-end reads of length 250 bp. The mosquito datasets [21] (pool20 and pool27) analyzed here originated from mosquito control districts throughout California. The resulting libraries were generated as previously described [43, 44], and sequenced with the HiSeq 4000 Illumina platform, using 2 × 150 cycle HiSeq. The Human metagenomic datasets characterize viral nucleic acids in nasal swabs or feces from apparently healthy young children with no recorded symptoms living in multiple small and remote Amazonian villages as described in our previous studies [13, 45]. The raw 250 bp paired-end reads were generated using MiSeq and deposited in Sequence Read Archive (SRA). The SRA project accession numbers are PRJNA391715 and PRJNA530270 for the mosquito samples and Amazon nasal swab samples respectively. The Amazon fecal samples are under accession numbers SRR6287056 to SRR6287135. We also included additional metagenome samples from human stool (47,210-feces) and human blood for a treated HIV positive sample (12-110034-veqrpcr), dog diarrhea stool (Dog-pool), and fish tumor tissue (Fish1-pool).

**Table 1:** Metagenome datasets used to evaluate ContigExtender performance

Data set	Sample	Read length	#reads	Genome type	Sequencing platform	Description
NIBSC	NIBSC-26	250	8.55 M	25 different human RNA and DNA viral pathogens	MiSeq	Multiplexed viral standards
Animal	Mosquito Pool20	150	0.81 M	Culex Iflavi-like virus Mesoniviridae	HiSeq4000	Mosquito pool
Animal	Mosquito Pool27	150	1.54 M	Culex Iflavi-like virus Mesoniviridae	HiSeq4000	Mosquito pool
Animal	Fish1-pool	250	2.30 M	Enterococcus virus	MiSeq	Fish tumor mass
Animal	Dog-pool	250	1.31 M	Uncultured crAssphage	MiSeq	Dog stool sample
Human	12-110034-veqrpcr	250	0.53 M	Hepacivirus C	Miseq	Human blood sample
Human	47210-feces	250	1.90 M	Escherichia virus	Miseq	Human stool sample
Human	Amazon-4B	250	0.81 M	Norwalk Virus	Miseq	Human stool sample
Human	Amazon-3D	250	0.38 M	Husavirus	Miseq	Human stool sample
Human	Amazon-17D	250	1.61 M	Husavirus	Miseq	Human stool sample
Human	Amazon-6D	250	0.47 M	Human Cosavirus	Miseq	Human stool sample
Human	Amazon-S10-CNI-055	250	0.95 M	Betapapillomavirus	Miseq	Human nasal swab sample

Genomic sequences from NIBSC, Animal and Human metagenome datasets represent various pathogen types, genome sizes, sample backgrounds, and sequencing outputs that were encountered in real world metagenome and clinical applications using NGS

### Preprocessing

Raw reads obtained from Illumina sequencing were preprocessed before assembly as described in [12]. Human host reads were subtracted by mapping the reads with human reference genome hg19 using bowtie2 [46]. Additionally, PRINSEQ version 0.20.4 was used to filter low complexity reads using default parameters [47].

### De novo assembly

We use SPAdes 3.13.0 with the “-meta” option to enable MetaSPAdes mode. The k-mer sizes were set to 21, 33, 55, and 77 while all other parameters were set to the default. metaSPAdes first constructs the de Bruijn graph of all reads using SPAdes, transforms it into the assembly graph using various graph simplification procedures, and reconstructs paths in the assembly graph that correspond to long fragments of individual genomes within a metagenome [24, 25]. Responding to the microdiversity challenge, metaSPAdes focuses on reconstructing a consensus backbone of a strain-mixture and thus sometimes ignores some strain-specific features (often corresponding to rare strains) to improve the contiguity of assemblies.

### Algorithm

**Fig. 1:**
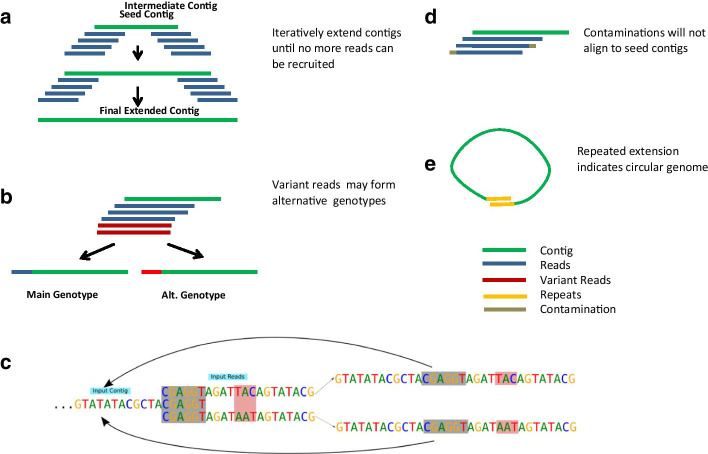
Schematic views of the ContigExtender assembly algorithm. (**a**) Iteratively recruit reads which overlap the edges of input contigs, then generate consensus sequence from the overlaps for form extended contigs. (**b**) Multiple strains may form alternative consensus contigs. Create branches when variant reads were detected. (**c**) A more detailed demonstration of the overlapping-consensus-branching algorithm, showing the two branches formed by depth first search (DFS). Two aligned reads have a three base disagreement region, so two different paths are formed for alternative extension. (**d**) Reads containing untrimmed adapters or other sequencing errors will not align well with contig and other reads. (**e**) Circular genome detection and extension termination

**Fig. 2:**
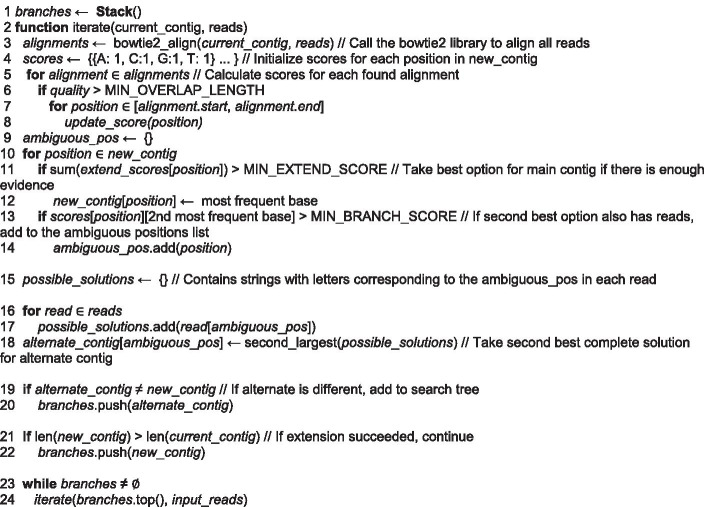
Pseudo code of ContigExtender algorithm

The outline of the algorithm is illustrated in Figs. 1 and 2. The efficacy of the extension results from individual optimization of each individual contig, instead of the simultaneous process used by de novo assembly. The extension process occurs iteratively. During each iteration, alignments between the set of reads and the two ends of the input contig are computed using an external tool such as Bowtie 2. After these alignments are filtered for quality and length, they are aggregated and regions with high disagreement are recorded. Then, each potential solution is scored and becomes one of multiple possible consensuses. Each then becomes the input contig of the upcoming extension iteration. These steps are repeated until a cycle is detected or insufficient alignments are available to extend the contig further. The final output of the algorithm consists of a collection of potential contigs.

#### Alignment

The algorithm utilizes an existing alignment tool to find overlaps between the reads and the contig. To preserve only partially overlapping reads, and not reads that lie completely within the contig, only the prefix and suffix of the contig with length equal to the length of the longest read are considered. The bowtie2 parameters are chosen to not penalize ambiguous characters when running end-to-end alignment. For paired data, we provide an option to constrain the orientation and distance of each read pair. When the constraints are enabled, reads whose mates are aligned discordantly are discarded.

#### Computation of consensus

For each overlap k, a quality score $q_{k}$ is computed, equal to the number of bases that the read and contig are in agreement on. Portions of the read that lie outside the original contig are not considered in this quality score. Each read must have a quality score above a certain user-defined threshold to be considered in the consensus, effectively ignoring short alignments of very few bases.

Let ${\text{R}}_{{\text{k}}} \left[ {\text{i}} \right]$ be the nucleotide of read k that would be at position i in the new consensus. At each position in the extended contig, define ${\text{Q}}\left[ {\text{i}} \right]\left[ j \right] = \sum\nolimits_{{R_{k} \left[ i \right] = jR_{k} \left[ i \right] = j}} {q_{k}^{2} }$. To create the new consensus c, set ${\text{c}}_{{\text{i}}}$ to be the base $j$ that maximizes $Q\left[ i \right]\left[ j \right]$ if this value is greater than a threshold. This threshold can be computed from the following expression:

$$threshold = 10^{ - t} l^{2} c,$$

where t is a dimensionless tolerance value that represents the acceptable amount of evidence required for extension, l is the length of the reads, and c is an estimate of the coverage.

If this threshold is not reached, then leave this position in the consensus undefined. This process computes the main consensus, which is supported by the most evidence.

#### Alternate consensuses

The second most highly scoring base for each position I is found, and if its score is greater than some threshold, and a sufficiently large number of reads agree on this, then position I is marked as an ambiguous base. For each read, a string is created by taking the subset of the read such that the chosen positions correspond with the ambiguous bases. These strings represent each individual read’s solution to the ambiguous positions. The frequencies of these strings are sorted and those meet a certain threshold of matching reads are retained as alternate consensuses.

To prevent the number of alternates from growing exponentially with the length of the contig, a limit may be set on the quantity of alternates that may be considered at each iteration, in addition to adjustment of the threshold required for designating a position as ambiguous.

The algorithm terminates when a sufficiently large prefix or suffix of the extended contig is identical to one that has occurred earlier in the extension process, as this would create an infinite loop of the exact alignments and consensuses that occurred in between. This also handles circular chromosomes, which would otherwise experience the same behavior. Additionally, an upper limit to the length of the extended contig can also be set, to save processing time when a consensus that diverges from the reference is chosen.

## Results

### Software parameters

The performance of ContigExtender on simulated and real datasets is benchmarked against the existing contig extension tools PRICE [32], Kollector [48] and GenSeed-HMM [49]. PRICE V 1.2 is executed using the recommended parameters: 30 cycles, 500 bp insert size, and 90% identity to the initial contig. Kollector is executed with the recommended alignment thresholds, assembly K-mer size 32, and overlap K-mer size 25. We ran GenSeed-HMM with the ABySS assembler, 30 bp extension seeds, and a 90% alignment threshold. ContigExtender is run with the default tolerance of 2.5.

### Simulated data

The performance of ContigExtender on the in silico synthetic BASV dataset is demonstrated in Table 2. From randomly chosen 1 kb seed contigs, ContigExtender was able to reconstruct nearly perfect genomes for all three viral genomes in all cases except for two challenging situations: (1) low sequencing depth 10 × coupled with short reads (100 bp) and (2) low depth 10 × coupled with high error rates (0.05). Results from the BKV and HIV1 datasets show similar trends (Additional file 1: Table S1). Although sequencing depth is a major factor for ContigExtender, longer reads of 250 bp coupled with low error rates (0.01) can compensate for low sequencing depth. ContigExtender can detect the circular nature of the BKV genome, avoiding over-extension.

**Table 2:** Comparison of contigs produced from in silico reads from the BASV virus

BASV (11.9 kb)			Error rate = 0.01					Error rate = 0.05				
Read length (bp)	Depth (x)	Spike-in peaks	Contig Extender (kb)	PRICE (kb)	GenSeed (kb)	Kollector (kb)	Meta SPAdes (kb)	Contig Extender (kb)	PRICE (kb)	GenSeed (kb)	Kollector (kb)	Meta SPAdes (kb)
100	10		6.7	1.6	1.1	1.8	6.7	1.9	1.3	NA	NA	1.8
	20		11.9	11.9	1.1	2.7	11.9	11.9	11.8	NA	NA	7.9
	20	Yes	11.9	11.8	1.1	2.7	11.9	11.9	11.9	NA	NA	7.9
	50		11.9	11.9	1.1	4.9	11.9	11.9	11.8	1.1	NA	7.9
250	10		11.8	7.2	1.1	1.1	7.9	8.4	7.3	NA	NA	7.6
	20		11.9	11.7	1.3	2.3	7.9	11.8	11.8	NA	NA	7.8
	20	Yes	11.9	11.5	1.3	2.3	7.9	11.8	NA	NA	NA	7.9
	50		11.9	11.8	1.4	2.4	7.9	11.9	11.9	NA	NA	11.9

Longest contig length produced by ContigExtender and other tools using in silico synthetic sequences for the BASV virus, representing varying read lengths, error rates, depths, and unevenness of coverage. Randomly selected sequences of 1 kb were used as seed contigs. Spike-in sequences were added, with each peak at a depth of at 50 × coverage and spanning 250 bp. De novo assemblies using metaSPAdes were also performed as a benchmark for assembly difficulty for each dataset. Runs that fail to produce extension are marked “NA”

MetaSPAdes is not directly comparable with ContigExtender since the former is a de novo assembler, whereas the latter is a seeded assembler. Nevertheless, MetaSPAdes results provide a benchmark to measure the difficulty of each dataset. Like ContigExtender, MetaSPAdes is sensitive to very low depth at 10x, but MetaSPAdes is less tolerant of sequencing errors and the existence of viral mixtures.

ContigExtender generally performed better than PRICE in low depth (10×) and high error rate datasets (Table 2 and Additional file 1: Table S1). Both reconstructed nearly the entire reference genome when given higher depth sequencing data. GenSeed-HMM and Kollector both reconstructed portions of the reference genome from low error rate reads but generally did not accomplish any extension in the high error rate datasets.

We also benchmarked ContigExtender on realistic paired-end simulated datasets generated by pIRS (Additional file 1: Table S2). The results suggest that ContigExtender outperforms PRICE at low coverage (10×). Additionally, while GenSeed-HMM and Kollector produced no output in some cases, ContigExtender produced extension in all trials. Also, we observed a clear advantage when ignoring the insert size constraint for paired-end extension. This allows the algorithm to overcome some difficult regions.

### NIBSC data

Among 58 MetaSPAdes seed viral contigs that are at least 1.5 kb in length and are at least 95% aligned to one of the reference viral genomes, 26 contigs were extended by at least 200 bp (Table 3). The quality of extended contigs was measured by final length, gained length (final length of ContigExtender output minus length of metaSPAdes seed contig), and the percentage of the output contig that is aligned to target viral genomes. The depth for each contig varies from 7× to 267×, the final contig lengths range from 1.7 to 10 kb, and the largest extension is 5.8 kb.

**Table 3:** ContigExtender results on NIBSC datasets using MetaSPAdes assembly outputs as seed contigs

Contig ID	Meta SPAdes (bp)	Contig Extender (bp)	Gained length (bp)	Gained (%)	Aligned (bp)	Aligned (%)	Viral genome (Accession)	Genome size (bp)	Gained genome (%)	PRICE (bp)	GenSeed (bp)	Kollector (bp)	Depth (x)
1	4251	10,059	5808	137	10,057	100	Human_rubulavirus_2 (NC_003443.1)	15,646	37				54
2	3114	8315	5201	167	8288	100	Human_mastadenovirus_C (NC_001405.1)	35,937	14				31
3	4705	6841	2136	45	6814	100	Human_mastadenovirus_C (NC_001405.1)	35,937	6				36
4	4118	5099	981	24	5057	99	Human_mastadenovirus_C (NC_001405.1)	35,937	3				36
5	2818	5063	2245	80	5062	100	Human_alphaherpesvirus_3 (NC_001348.1)	124,884	2				14
6	2234	4671	2437	109	4675	100	Human_betaherpesvirus_5 (NC_006273.2)	235,646	1				28
7	1784	4224	2440	137	4224	100	Human_alphaherpesvirus_3 (NC_001348.1)	124,884	2				11
8	3944	4171	227	6	4149	99	Human_mastadenovirus_C (NC_001405.1)	35,937	1				29
9	3051	4098	1047	34	4092	100	Human_alphaherpesvirus_3 (NC_001348.1)	124,884	1				16
10	3158	4029	871	28	3575	89	Human_betaherpesvirus_5 (NC_006273.2)	235,646	0				42
11	3462	3964	502	15	3961	100	Human_alphaherpesvirus_3 (NC_001348.1)	124,884	0				23
12	1789	3666	1877	105	3665	100	Human_alphaherpesvirus_3 (NC_001348.1)	124,884	2				14
13	1761	3379	1618	92	3319	98	Rotavirus_A (NC_011507.2)	3302	49	3403			126
14	1759	3292	1533	87	2552	78	Bat_rotavirus (NC_040413.1)	2649	58	2274		2101	267
15	2748	3140	392	14	3146	100	Human_betaherpesvirus_5 (NC_006273.2)	235,646	0				24
16	2861	3115	254	9	3115	100	Human_respirovirus_1 (NC_003461.1)	15,600	2				29
17	2664	3016	352	13	3016	100	Human_mastadenovirus_C (NC_001405.1)	35,937	1				25
18	1525	2839	1314	86	2840	100	Human_alphaherpesvirus_3 (NC_001348.1)	124,884	1				7
19	1958	2616	658	34	2612	100	Human_alphaherpesvirus_3 (NC_001348.1)	124,884	1				27
20	1789	2213	424	24	2213	100	Human_alphaherpesvirus_3 (NC_001348.1)	124,884	0				8
21	1889	2154	265	14	2154	100	Human_betaherpesvirus_5 (NC_006273.2)	235,646	0				29
22	1881	2093	212	11	2093	100	Human_alphaherpesvirus_3 (NC_001348.1)	124,884	0				19
23	1748	2001	253	14	1996	100	Human_alphaherpesvirus_3 (NC_001348.1)	124,884	0				14
24	1699	1931	232	14	1931	100	Human_alphaherpesvirus_3 (NC_001348.1)	124,884	0				21
25	1505	1846	341	23	1847	100	Human_betaherpesvirus_5 (NC_006273.2)	235,646	0				27
26	1508	1768	260	17	1770	100	Human_betaherpesvirus_5 (NC_006273.2)	235,646	0				26

Columns 2–14 are: 2) seed contig length generated by MetaSPAdes; 3) extended contig length from seed contig; 4) gained length from ContigExtender (column 3 subtracted by column 2); 5) gained length as percentage of seed contig (column 4 divided by column 2); 6) the largest contiguous segment length of extended contig that are aligned to reference genome; 7) percentage of the alignment segment of the extend contig; 8) reference viral genome; 9) viral genome size; 10) gained length from ContigExtender as percentage of viral genome (column 4 divided by column 9); 11) gained extension by PRICE; 12) gained extension by GenSeed; 13) gained extension by Kollector; 14) average sequencing depth of the extended contig. Entries in the PRICE, GenSeed, and Kollector columns are blank if they produced no extension

The accuracy of the final contigs is measured by the quality of alignment of these contigs to their respective reference viral genomes. Out of the 26 final contigs, all but two have nearly perfect alignments to reference genomes with greater than 98% single segment alignment. The other 2 contigs, Contig 10 (89%) and Contig 14 (78%) do not have a single alignment covering the whole contig, indicating possible chimeric contig formation during the contig extension process. Note that the performance reported here is achieved using ContigExtender’s default scoring parameters which can be adjusted to be more aggressive or more conservative. Aggressive extension produces longer contigs but risks higher chances of chimeric contig formation, while conservative extension results in shorter but possibly more accurate contigs.

PRICE, GenSeed-HMM, and Kollector did not produce any extension of most of the seed contigs tested (Table 3).

**Fig. 3:**
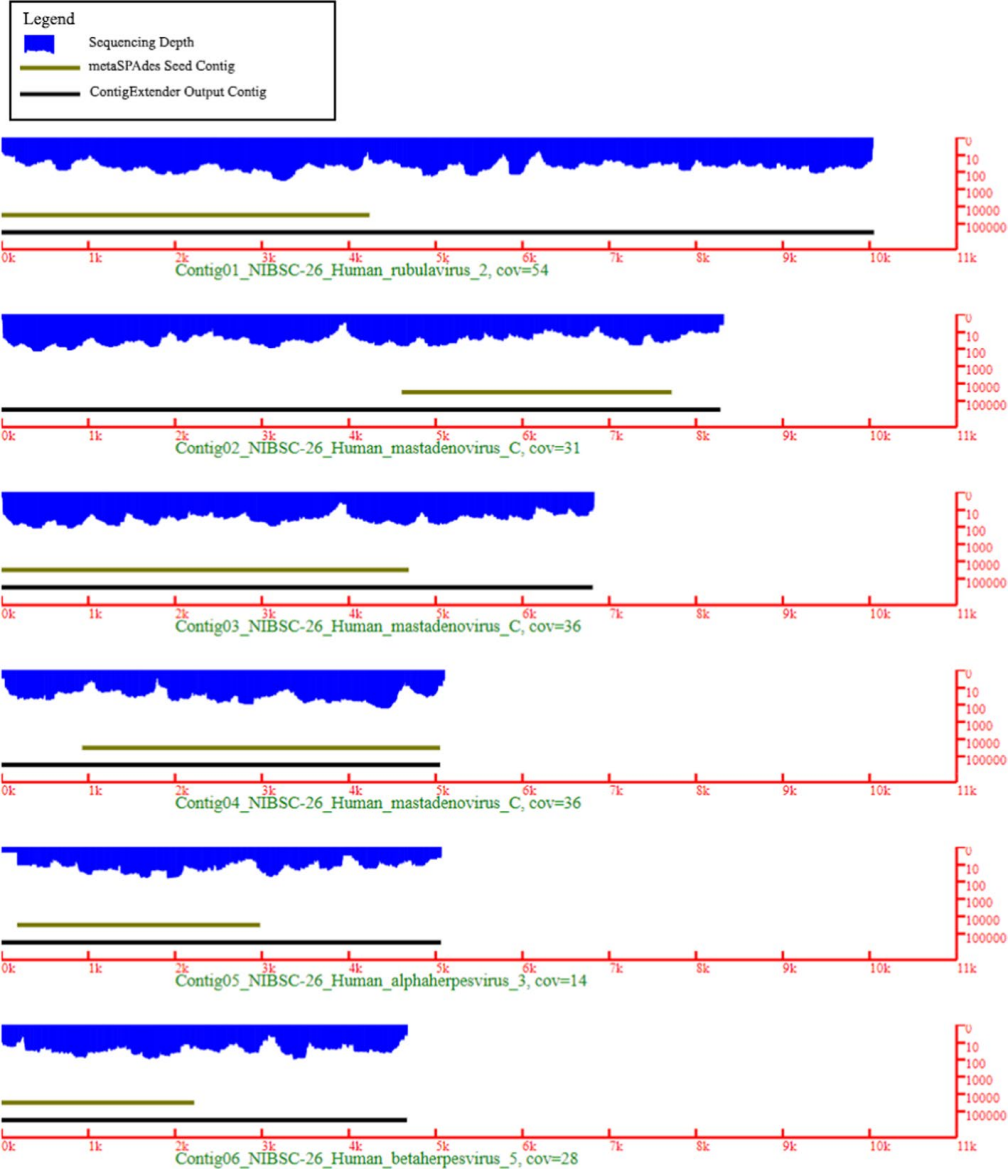
ContigExtender output shown alongside metaSPAdes seed contig and sequencing depth. Reads mapped to the final contig shown as wiggle plots (in blue), seed contigs generated by MetaSPAdes (dark brown line), and final contig regions that are aligned to reference viral genome (black line). The y axis is the depth in log scale and x axis is the contig length. This figure were generated from native Scalable Vector Graphics (SVG) images plotted using Python 3 scripts based on reads mapping to the viral reference genomes with blastn

Figure 3 shows the wiggle plot of the top 6 longest final contigs for the NIBSC dataset. Contig2 and Contig3 wiggle plots are highly similar; they are variants of the same contig that aligned to the same region of Human Mastadenovirus C. The coverage is very uneven within the regions of each contig and across different contigs. Not surprisingly, the low coverage valleys are one of the main reasons that the assembly terminates prematurely. A sudden dip in the coverage will likely end the contig assembly or extension.

### Human and animal data

Ten contigs achieved significant extensions of greater than 0.2 kb from the viral seed contigs that are longer than 1.5 kb in the “Animal” dataset. Contig 5 is the only contig that has a significant chimeric extension of ~ 3 kb, but it still gained a ~ 4 kb correct extension (Table 4). For the “Human” dataset, 13 contigs achieved significant extension and were successfully aligned to a wide variety of reference viruses. The only contig that has a possible chimeric extension is Contig 1 (Table 5) which gained a ~ 2.4 kb extension that does not align to the Norwalk virus reference genome. All other contigs are successful extensions and some gained significant length, as much as 6 kb, such as Contig 3 from sample Amazon-17D.

**Table 4:** ContigExtender results on Animal datasets using MetaSPAdes as seed contigs

Contig ID	Library	Meta SPAdes (bp)	Contig Extender (bp)	Gained length (bp)	Gained (%)	Aligned (bp)	Aligned (%)	Viral genome	Genome size (bp)	Gained genome (%)	PRICE (bp)	GenSeed (bp)	Kollector (bp)
1	Dog-pool	5521	9826	4305	78	9760	99	uncultured_crAssphage (NC_024711.1)	97,065	4			
2	Fish1-pool	2723	7064	4341	159	6878	97	Enterococcus_virus_phiSHEF5 (NC_042023.1)	41,598	10			
3	Mosquito-pool20	3074	10,130	7056	230	9699	96	Culex_Iflavi-like_virus_4 (NC_040716.1)	9698	73			
4	Mosquito-pool20	3042	10,130	7088	233	9699	96	Culex_Iflavi-like_virus_4 (NC_040716.1)	9698	73			
5	Mosquito-pool27	4106	10,095	5989	146	7030	70	Culex_Iflavi-like_virus_4 (NC_040716.1)	9698	62			
6	Mosquito-pool27	6011	10,069	4058	68	10,068	100	Alphamesonivirus_1 (NC_015874.1)	20,192	20			6742
7	Mosquito-pool27	5638	10,016	4378	78	9673	97	Culex_Iflavi-like_virus_4 (NC_040574.1)	9698	45	7820		
8	Mosquito-pool20	3689	9872	6183	168	9699	98	Culex_Iflavi-like_virus_4 (NC_040716.1)	9698	64			
9	Mosquito-pool27	2430	2674	244	10	2626	98	Culex-associated_Tombus-like_virus (NC_040575.1)	2645	9			
10	Mosquito-pool27	1786	2131	345	19	2052	96	Hubei_mosquito_virus_4 (NC_032231.1)	4971	7			

Columns 3–14 are: 3) seed contig length generated by MetaSPAdes; 4) extended contig length from seed contig; 5) gained length from ContigExtender (column 4 subtracted by column 3); 6) Gained length as percentage of seed contig (column 5 divided by column 3); 7) the largest contiguous segment length of extended contig that are aligned to reference genome; 8) percentage of the alignment segment of the extend contig; 9) reference viral genome; 10) viral genome size; 11) gained length from ContigExtender as percentage of viral genome (column 5 divided by column 10); 12) gained extension by PRICE; 13) gained extension by GenSeed; 14) gained extension by Kollector. Entries in the PRICE, GenSeed, and Kollector columns are blank if they produced no extension

**Table 5:** ContigExtender results on Human metagenome datasets using MetaSPAdes as seed contigs

Contig ID	Library	Meta SPAdes (bp)	Contig Extender (bp)	Gained length (bp)	Gained (%)	Aligned (bp)	Aligned (%)	Genome	Genome size (bp)	Gained genome (%)
1	Amazon-4B	7560	10,034	2474	33	7493	75	Norwalk_virus (NC_040876.1)	7521	33
2	Amazon-17D	7912	8329	417	5	7862	94	Husavirus_sp. (NC_032480.1)	8856	5
3	Amazon-3D	1537	7676	6139	399	7678	100	Husavirus_sp. (NC_032480.1)	8856	69
4	Amazon-3D	3776	7530	3754	99	7532	100	Husavirus_sp. (NC_032480.1)	8856	42
5	Amazon-3D	2165	7530	5365	248	7532	100	Husavirus_sp. (NC_032480.1)	8856	61
6	Amazon-S10-CNI-055	1671	3258	1587	95	3242	100	Betapapillomavirus_1 (NC_001531.1)	7746	20
7	Amazon-S10-CNI-055	1710	3258	1548	91	3242	100	Betapapillomavirus_1 (NC_001531.1)	7746	20
8	Amazon-6D	2151	2772	621	29	2681	97	Human_cosavirus (NC_023984.1)	7802	8
9	12-110034-veqrpcr	2339	5237	2898	124	5233	100	Hepacivirus_C(NC_004102.1)	9646	30
10	47210-feces	2436	4637	2201	90	4444	96	Escherichia_virus_AKFV33 (NC_017969.1)	108,853	2
11	47210-feces	2436	3572	1136	47	3572	100	Escherichia_virus_T5 (NC_005859.1)	121,750	1
12	12-110034-veqrpcr	2424	3157	733	30	3121	99	Hepacivirus_C (NC_004102.1)	9646	8
13	12-110,034-veqrpcr	2424	3156	732	30	3121	99	Hepacivirus_C (NC_004102.1)	9646	33

Columns 3–11 are: 3) seed contig length generated by MetaSPAdes; 4) extended contig length from seed contig; 5) gained length from ContigExtender (column 4 subtracted by column 3); 6) Gained length as percentage of seed contig (column 5 divided by column 3); 7) the largest contiguous segment length of extended contig that are aligned to reference genome; 8) percentage of the alignment segment of the extend contig; 9) reference viral genome; 10) viral genome size; 11) gained length from ContigExtender as percentage of viral genome (column 5 divided by column 10). Note that PRICE, GenSeed, and Kollector did not extend any seed contigs in this set, so their columns are omitted

For the combined 49 contigs from the three human and animal datasets, 45 are of high quality without chimeric extensions. For these 45 contigs, the average seed contig length generated by MetaSPAdes is 2.8 kb. ContigExtender increased these lengths by ~ 2 kb on average, resulting in a final average length of 4.8 kb. The median gained length, however, is ~ 1.5 kb and the median seed and output lengths are 2.4 kb and 3.9 kb, respectively. These results, shown in Tables 3, 4, and 5, demonstrate a significant improvement over de novo assembly in a wide variety of datasets. In comparison, PRICE and Kollector successfully extended only one contig each, while GenSeed-HMM extended zero contigs.

## Discussions

Genome sizes of bacteriophages and viruses range from a few kb to several hundred kb. State-of-the-art de novo assemblers can only achieve contigs that are a few kb long; these contigs can often be further extended by iteratively mapping reads to the contig ends, which is currently a time consuming, manual process. The proposed method effectively turns the sequence assembly process into a two-step process: de novo assembly followed by contig extension. Our results demonstrated that contig extension can be an effective step in improving metagenomic sequence analysis. Compatible with any de novo assembler, ContigExtender can be built into most viral metagenomics analysis pipelines. A wide range of metagenomic applications such as pathogen detection, microbiological surveillance and viral discovery, can benefit from contig extension, which significantly reduces the time and effort required for manual contig extension.

A feature to combat the microdiversity challenge is the novel branching feature proposed by ContigExtender. The proposed DFS branching mechanism allows multiple branching paths, defined by different genotypes representing multiple strains. Each overlapping region is evaluated for homogeneity of read alignments. Potential branch points are created at the overlapping region when sufficient heterogeneity, representing multiple genotypes (strains) is observed. Intro- and inter-genomic repeats can also be genotyped and resolved during this branching, which allows the exploration of multiple extension paths to gain maximum extension.

Two major factors causing premature assembly termination are abrupt dips in coverage and excessive sequencing errors and contaminations. The former is observed in Fig. 3, which shows that many contigs failed at coverage valleys. The latter is observed in Table 2 where excessive sequencing error (5%) causes poor de novo contig formation on many of the silico synthesized datasets. ContigExtender addresses these challenges by utilizing a novel extension scoring function prioritizing overlapping over depth. It focuses on overlap quality rather than using a hard depth cutoff for extension on low coverage regions. To avoid chimeric contig extension as much as possible, our scoring function requires increased overlap lengths for low coverage regions. By using alignment rather than the kmer search utilized in most de novo assemblers, ContigExtender trades speed for accuracy, allowing for better performance in high sequencing error regions.

These features may explain the favorable performance of ContigExtender relative to other contig extension tools. PRICE iteratively assembles proximal reads and fills gaps between contigs using paired-end relationships. Kollector recruits reads using progressive Bloom filters instead of alignment. GenSeed-HMM, in a similar process to ContigExtender, iteratively finds similar reads and extends contigs through assembly software. These tools have a common element in that they all utilize de Bruijn assemblers to generate a consensus sequence. When the input contig is the final output of a de novo assembly tool such as metaSPAdes, further assembly based on de Bruijn graphs is unlikely to succeed, as the factors which caused metaSPAdes to terminate extension remain in the data. Thus, computing the consensus sequence using the scoring function employed by ContigExtender is more likely to overcome these challenging regions.

The viral reference genome database is by no means a gold standard for evaluating contigs because there are still many unknown viral species and strains not represented in the database. Therefore, some extended contigs cannot be aligned either to their originating genome or to a distant genome, and thus are designated as false chimeric contigs in our analysis. The NIBSC data, however, can be evaluated more accurately, because the samples only include known virus standards.

As we have shown in Tables 3, 4, and 5, there are several extensions that are the results of over-assembly or mis-assembly, as indicated by less than 100% alignment to reference genomes. We also observed multiple assemblies covering the same genomic regions, due to either sequencing errors or the mixtures of multiple strains in metagenomic samples. Mis-assembly can happen quite often in the initial de-novo assembly for generating seed contigs and it is even more likely in contig extension because contig extension pushes the limit of contig lengths by accepting a higher risk for mistakes. Our algorithm’s scoring system favors the most probable extensions and the balance between extension length and the probability of mis-assembly is adjustable. In practice, contig extension mis-assemblies or chimeric contigs can be identified when aligning extended contigs against known viral genomes, as we show in the results (Fig. 3 and Tables 3, 4, 5). For novel viruses, the identification of chimeric contigs cannot be achieved computationally but it can be accomplished through PCR extension. All the mis-assemblies in our tests (Tables 3, 4, 5) are segments from the same genome. After careful examinations of these mis-assemblies against reference genomes, we found that these mis-assemblies are caused by extending contig ends with incorrect reads from a different region of the same genome.

The current software is only optimized and tested on viral metagenomes, not for bacterial or eukaryotic genomes. We speculate that our current version may not work well for other genomes for two reasons: 1) Viral genomes contain considerably fewer repeats than other genomes; and 2) the sequencing dataset sizes for non-viral genomes are usually considerably larger, so the running time may require further optimization.

## Conclusions

We have presented a new approach for enhancing the performance of de novo metagenomics assemblers. The proposed DFS branching algorithm allows multiple branching paths defined by different genotypes representing multiple strains. Our strategy automates the labor-intensive process of manually constructing viral genomes from the fragments produced by de novo assembly. With simulated and real-world animal and human metagenomics datasets, ContigExtender is demonstrated to be effective in improving upon both de novo assembly alone and de novo assembly combined with other extension tools. For contigs that are extendable, ContigExtender can accurately increase the contig length by several kb, which is significant for viral genomes. The software may also be incorporated into viral metagenomics analysis pipelines, with a variety of applications such as pathogen detection, viral discovery, clinical microbiology and environmental metagenomics. Thus, we believe that the use of our software will be of broad interest to researchers, epidemiologists, clinicians, and environmental biologists.

## Availability and requirements

- Project name: ContigExtender
- Project home page: https://github.com/dengzac/contig-extender
- Operating system(s): Linux, Windows
- Programming language: Python 3.6 or higher
- Other requirements: Bowtie2 2.3.5 or higher, Perl 5 or higher
- License: GNU GPLv3
- Any restrictions to use by non-academics: None

## Supplementary Information

**Additional file 1: Supplementary Table S1**. shows a comparison of contigs produced by ContigExtender, PRICE, Kollector, GenSeed, and metaSPAdes from in silico reads of the BKV and HIV viruses. **Supplementary Table S2** shows contig lengths produced by ContigExtender on simulated paired-end reads from pIRS (read length 100, error rate 0.05).

## Data Availability

The datasets analyzed during the current study are available in the Sequence Read Archive repository, at https://www.ncbi.nlm.nih.gov/sra. Accession numbers are PRJNA391715 and PRJNA530270 for mosquito samples and Amazon nasal swab samples respectively. The Amazon fecal samples are under accession numbers SRR6287056 to SRR6287135.

## Abbreviations

mNGS: Metagenomic Next-Gen Sequencing
DFS: Depth-First Search
