# Supplementary material for: ContigExtender: a new approach to improving de novo sequence assembly for viral metagenomics data
Source: BMC Bioinformatics. 2021 Mar 12;22:119. doi: 10.1186/s12859-021-04038-2 (PMC7953547; doi:10.1186/s12859-021-04038-2)
Supplement: Supplementary file 1 — Additional file 1: Supplementary Table S1. shows a comparison of contigs produced by ContigExtender, PRICE, Kollector, GenSeed, and metaSPAdes from in silico reads of the BKV and HIV viruses. Supplementary Table S2 shows contig lengths produced by ContigExtender on simulated paired-end reads from pIRS (read length 100, error rate 0.05). [file 12859_2021_4038_MOESM1_ESM.docx]

Supplementary Table 1: Comparison of contigs produced by ContigExtender, PRICE, Kollector, GenSeed, and metaSPAdes from *in silico* reads of the BKV and HIV viruses. Runs that fail to produce extension are marked “NA”.

|  | | | | Error Rate = 0.01 | | | | | Error Rate = 0.05 | | | | |
| --- | --- | --- | --- | --- | --- | --- | --- | --- | --- | --- | --- | --- | --- |
| Virus  (Genome) | Read  Length  (bp) | Depth  (x) | Spike-in  peaks | Contig  Extender  (kb) | PRICE  (kb) | GenSeed  (kb) | Kollector  (kb) | Meta  SPAdes  (kb) | Contig  Extender  (kb) | PRICE  (kb) | GenSeed  (kb) | Kollector  (kb) | Meta  SPAdes  (kb) |
| BKV  (5.2kb) | 100 | 10 |  | 2.8 | 3.6 | NA | 2.0 | 2.8 | 4.5 | 4.5 | NA | NA | 3.5 |
|  |  | 20 |  | 4.8 | 4.8 | 1.2 | 3.4 | 4.9 | 4.9 | 4.8 | NA | NA | 4.9 |
|  |  | 20 | yes | 4.9 | 4.8 | 1.2 | 3.4 | 4.9 | 3.9 | 4.8 | NA | NA | 4.9 |
|  |  | 50 |  | 4.9 | 4.8 | 1.2 | 4.4 | 4.9 | 4.8 | 4.8 | 1.1 | NA | 4.9 |
|  | 250 | 10 |  | 4.7 | 4.0 | NA | NA | 4.9 | 4.9 | 2.7 | NA | NA | 3.1 |
|  |  | 20 |  | 4.8 | 5.0 | 1.2 | NA | 4.9 | 5.1 | 4.8 | NA | NA | 4.9 |
|  |  | 20 | yes | 5.0 | 5.1 | 1.2 | NA | 4.9 | 5.1 | 5.1 | NA | NA | 4.9 |
|  |  | 50 |  | 4.9 | 5.1 | 1.4 | 3.3 | 4.9 | 5 | 5.1 | NA | NA | 3.2 |
| HIV1  (9.2kb) | 100 | 10 |  | 5.2 | 2.4 | 1.2 | 1.6 | 5.2 | 1.4 | 1.0 | NA | NA | 2.7 |
|  |  | 20 |  | 8.7 | 8.6 | 1.2 | 2.7 | 6.3 | 8.5 | 8.6 | NA | NA | 5.1 |
|  |  | 20 | yes | 8.6 | 8.9 | 1.2 | 2.7 | 4.8 | 8.8 | 8.6 | NA | NA | 7.9 |
|  |  | 50 |  | 8.6 | 8.9 | 1.4 | 4.8 | 8.5 | 8.6 | 8.9 | NA | NA | 5.1 |
|  | 250 | 10 |  | 8.9 | 6.7 | 1.3 | NA | 7.4 | 6.5 | 1.6 | NA | NA | 3.3 |
|  |  | 20 |  | 8.8 | 9.0 | 1.3 | 2.9 | 7.4 | 9.1 | 9.1 | NA | NA | 4.8 |
|  |  | 20 | yes | 8.6 | 8.5 | 1.3 | NA | 5.1 | 9.1 | 9.0 | NA | NA | 7.1 |
|  |  | 50 |  | 8.6 | 9.0 | 1.4 | 3.6 | 5.5 | 8.8 | 9.1 | 1.2 | 1.2 | 8.7 |

Supplementary Table 2: Contig lengths produced by ContigExtender on simulated paired-end reads from pIRS (read length 100, error rate 0.05). Runs that fail to produce extension are marked “NA”.

| Genome, genome size  (Coverage) | PRICE (kb) | GenSeed  (kb) | Kollector  (kb) | ContigExtender  (kb, using insert size constraint) | ContigExtender (kb, ignoring insert size constraint) |
| --- | --- | --- | --- | --- | --- |
| BASV, 11.9kb (cov=10) | 0.8 | NA | NA | 1.8 | 1.9 |
| BASV (cov=20) | 7.8 | 1.1 | 4.4 | 5.6 | 8.1 |
| BKV, 5.2kb (cov=10) | 0.8 | 1.1 | NA | 2.8 | 3.0 |
| BKV (cov=20) | 4.9 | 1.1 | NA | 5.0 | 5.1 |
| HIV1, 9.2kb (cov=10) | 0.7 | NA | 1.6 | 2.7 | 2.3 |
| HIV1 (cov=20) | 6.8 | 1.1 | 6.3 | 7.3 | 9.4 |
